# The Role of Computed Tomographic Angiography in Predicting the Development of Vasospasm Following Ruptured Intracranial Aneurysm Microsurgery

**DOI:** 10.7759/cureus.45386

**Published:** 2023-09-16

**Authors:** Eyüp Varol

**Affiliations:** 1 Neurological Surgery, Umraniye Training and Research Hospital, Istanbul, TUR

**Keywords:** computed tomographic angiography, vasospasm, hemorrhage, subarachnoid, aneurysm

## Abstract

Introduction

Following subarachnoid hemorrhage, cerebral vasospasm is the primary cause of morbidity and death. The aim of this study is to predict the development of vasospasm by detecting changes in vessel diameter after surgery using computed tomography angiography.

Methods

We retrospectively evaluated the patients who underwent aneurysm clipping due to a bleeding aneurysm between 2019-2022. Age, gender, location, subarachnoid hemorrhage grades, development of perioperative rupture, and temporary clip use were examined. Preoperative and postoperative diameters of the internal carotid artery, A1-A2, and M1-M2 were measured. Radiological and clinical vasospasm development in the postoperative period was also documented.

Results

The aneurysm localizations of the 100 patients (mean age: 50.38±13.04 years) were anterior cerebral artery in 50 patients, internal carotid artery in 37 patients, and middle cerebral artery in 30 patients. In the postoperative follow-up, radiological vasospasm was apparent in 41 patients. The changes in arterial diameter reveal a statistically significant decrease in the internal carotid artery, M1-M2, and A1-A2 artery diameters on the operated side compared to the contralateral side (p<0.001). Based on the receiver operating characteristic (ROC) analysis, the most likely change in arterial diameter on the operated side to indicate the presence of vasospasm was calculated from the available data, where the decrease in total arterial diameter was 13.7%.

Conclusion

Vasospasm remains one of the significant causes of morbidity and mortality post subarachnoid hemorrhage. While there have been advances in imaging modalities, predicting which patients will develop vasospasm has remained elusive. Our research attempts to provide a quantifiable metric (13.7% decrease in vessel diameter) that can be an early predictor of this complication.

## Introduction

Aneurysmal subarachnoid hemorrhage (aSAH) is a life-threatening disease that affects three to 25 people per 100,000 worldwide every year [1]. Vasospasm is evident in around 40% of aSAH patients, and 20-30% of aSAH patients suffer from vasospasm-related neurological impairments [2-5]. Following subarachnoid hemorrhage (SAH), cerebral vasospasm (CVS) is one of the common causes of morbidity and death [6]. Vasospasm affects 50-70% of SAH patients, with 50% of these patients experiencing neurological symptoms (i.e., symptomatic CVS) [7]. A new focal neurological deficit that is not explained by rebleeding or hydrocephalus and an altered level of consciousness are common indications of vasospasm. CVS is well known to cause neurological impairments through delayed cerebral ischemia [3,8,9].

Cerebral infarction affects half of all symptomatic CVS patients and is deadly in 30% of cases [10]. It is critical to research CVS to support the development of effective therapies and reduce the morbidity rate of people with this ailment. Despite efforts to develop novel medicines to prevent and cure CVS, it continues to be a major cause of death and mortality in patients who survive initial aSAH therapy [11,12]. One of the objectives of critical care monitoring in these patients is early diagnosis of CVS. Cerebral digital subtraction angiography (DSA) is currently the gold standard for diagnosing CVS [13,14]. Nevertheless, it is not apparent if these data might be comparable to the DSA resolution images, which are the gold standard imaging technique [15]. Magnification, the distance from the source of the image to the detector, and the viewing angle are some of the variables that influence vessel diameter measurements on DSA. Furthermore, measurements are frequently provided only in relative units when assessing pictures with simple imaging viewers [16].

Transcranial Doppler (TCD) ultrasonography is one example of the non-invasive methods that can be used to detect vasospasm in patients with aSAH. However, its low specificity and high operator dependence render it an inadequate detection tool for vasospasm [17]. Another non-invasive imaging technique, computed tomographic angiography (CTA), shows greater promise as a more accurate tool for evaluating vasospasm. This established routine screening tool provides non-invasive information about cerebral artery diameters [18]. Multidetector CTA, which enables rapid image acquisition and lower radiation exposure, has gained popularity over the last decade. Our aim in this study is to use CTA to evaluate changes in vessel diameter after surgery and assess the effects of those changes on vasospasm in order to predict the development of vasospasm.

## Materials and methods

Patient data and outcome assessment

For this research, we retrospectively evaluated the collected data of patients in our clinic who underwent aneurysm clipping due to a bleeding aneurysm between 2019 and 2022.

Inclusion criteria

The inclusion criteria included patients who underwent aneurysm clipping due to a bleeding aneurysm between 2019 and 2022; patient age greater than 18 years and less than 85 years; availability of both preoperative and immediate postoperative CTA and diffusion-weighted images (DWI) data; patients with complete clinical documentation, including recorded data about age, gender, aneurysm location, Fisher classification, Hunt-Hess (HH) classification, and World Federation of Neurosurgical Societies (WFNS) grade; and patients who provided written informed consent.

Exclusion criteria

The exclusion criteria included patients with incomplete clinical documentation or missing preoperative or postoperative imaging data; patients with other cerebral pathologies such as tumors, arteriovenous malformations, or previous strokes which could interfere with accurate measurement of arterial diameters; patients who did not provide informed consent or those unable to provide consent due to incapacitation; patients with other surgical interventions or endovascular procedures on the cerebral arteries within the previous year; patients with contraindications to CTA or DWI such as severe allergy to contrast agents or metallic implants; pregnant women or those nursing; and patients with chronic kidney disease or renal dysfunction, which contraindicates the use of contrast agents.

The recorded data concerns the patient's age and gender, the location of the aneurysm causing subarachnoid bleeding, Fisher classification, Hunt-Hess (HH) classification, WFNS grade, single or multiple aneurysm, development of perioperative rupture, and temporary clip use and duration. The recorded blood volumes in the aspirator were used to determine the amount of bleeding during the operation. Preoperative and postoperative vessel diameters of the internal carotid artery (ICA), A1-A2, and M1-M2 were measured on the operated and non-operated sides and noted. All measurements were made by two experienced neurosurgeons, and their mean values were taken. The development of radiological and clinical vasospasm in the postoperative period was also documented. If a newly developed restriction was observed on DWI, it was evaluated as a radiological vasospasm. It was accepted as a clinical vasospasm if a newly onset neurological deficit was observed. Patient data are reported according to common descriptive statistics. Written informed consent was obtained from all patients, and the study was performed in accordance with the ethical standards of the Declaration of Helsinki and under the approval of our institutional review committee. Ethical approval for the study was obtained from the Umraniye Training and Research Hospital Ethics Committee.

Radiological technique

**Figure 1:**
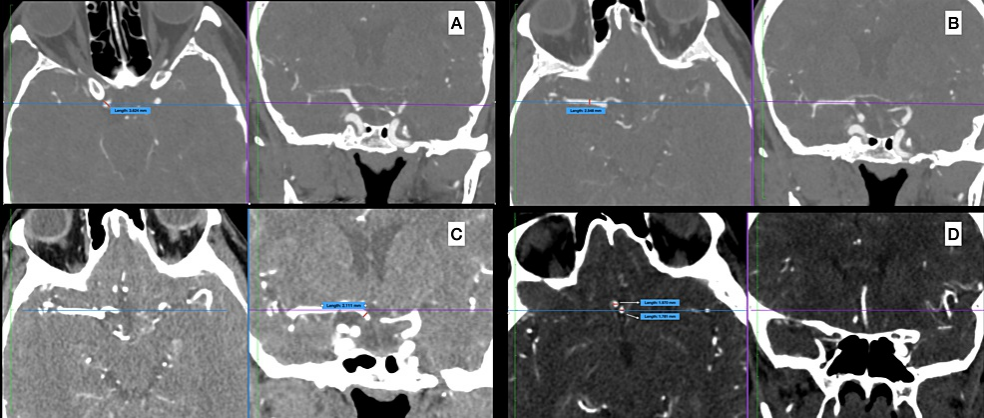
Measurements of arterial diameters in a 3D image viewer software a) internal carotid artery, b) M1 segment of middle cerebral artery, c) A1 segment of anterior cerebral artery, d) A2 segment of anterior cerebral artery

The same tomography device was employed for all patients in the study. Preoperative and postoperative vessel diameters were measured in all patients by two senior neurosurgeons, and the assessment used the average of the measurements taken by the two neurosurgeons. Preoperative and immediate (<24 hours) postoperative CTA and DWI were conducted according to a standard predefined imaging protocol. The vascular diameters were assessed in preoperative and postoperative CTA images on operated and non-operated parts. Measurements were performed at 1 cm distal to the anterior cerebral artery (ACA) origin, 1 cm distal to the M1 and M2 origins, and 1 cm proximal to the formation of the ACA and middle cerebral artery (MCA) composition for the ICA (Figure 1).

Preoperative and postoperative DWI were performed in all patients for radiological evaluation of vasospasm.

Statistical analysis

The Statistical Package for the Social Sciences (SPSS) version 25.0 (IBM Inc., Armonk, New York) program was used for statistical analysis to evaluate the results of this study. Descriptive, graphical, and statistical methods were applied to determine whether the scores obtained from each continuous variable were normally distributed. The Kolmogorov-Smirnov test was used to test the normality of the scores obtained from a continuous variable with the statistical method. Descriptive statistical methods (number, percentage, mean, median, standard deviation, etc.) were used while evaluating the research data. Comparisons between two groups in quantitative data were made with the independent samples t-test (in data with normal distribution) and Mann-Whitney U test (in data with no normal distribution), and comparisons of more than two groups were made with the Kruskal-Wallis test. The Bonferroni test was used to determine from which groups the difference originated, while Chi-squared tests (Pearson's chi-squared test, continuity correction test, and Fisher's exact test) were applied for qualitative comparisons between groups. Repeated measurements were made with the paired samples t-test. Receiver operating characteristic (ROC) analysis was used to determine the most appropriate rate of change in arterial diameter in the presence of vasospasm. A p-value of less than 0.05 was considered statistically significant with a 95% confidence interval.

## Results

Within the scope of the study, the findings of 100 patients were analyzed. The mean age of the patients was 50.38 ± 13.04 years, and 50% of them were in the age group of 50 years and above. Of the 100 patients, 51% were female, and 49% were male. According to radiological imaging results, the aneurysm localization was ACA in 50 patients, ICA in 37 patients, and MCA in 30 patients. Aneurysm was observed as multiple in 16 patients and single in 84 patients. Temporary clips were applied to 47% of the patients. The presence of perioperative rupture was reported in 34% of the patients, and the mean amount of perioperative bleeding was 306.20 ± 191.92 mL. In the postoperative follow-up, radiological vasospasm was apparent in 41 patients. During the treatment period, morbidity was observed in 15 (15%) patients, and mortality was observed in 13 (13%) patients (Table 1).

**Table 1:** Patient demographics (N=100) ACA - anterior cerebral artery, ICA - internal carotid artery, MCA - middle cerebral artery, Min - minimum, Max - maximum, SD - standard deviation

		n=%	Mean(SD)	Min.-Max.
Age	Total	100	50.38 (13.04)	24-79
Age group	<50	50		
	≥50	50		
Gender	Male	49		
	Female	51		
Surgery side	Right	55		
	Left	45		
Aneurysm location	ACA	50		
	ICA	37		
	MCA	30		
Aneurysm single/ multiple	Single	84		
	Multiple	16		
Temporary clip	Yes	47		
	No	53		
Perioperative rupture	Yes	34		
	No	66		
Vasospasm	Yes	41		
	No	59		
Perioperative bleeding (cc)	Total	100	306.20 (191.92)	50-1000
Discharge situation	Discharged with healing	72		
	Need care	15		
	Dead	13		

We examined the WFNS, Fisher, and HH scale results of the patients and found that the mean scores were 2.02 ± 1.30, 2.59 ± 1.19, and 2.12 ± 1.17, respectively. 

There was no statistically significant difference between the measurements made by the two neurosurgeons. The changes in arterial diameter reveal a statistically significant decrease in the ICA, M1-M2, and A1-A2 artery diameters on the operated side compared to the contralateral side (p<0.001). The evaluation of all arteries together found a 13.2% decrease in mean arterial diameter on the operated side and a 3% decrease on the opposite side (p<0.001). In the preoperative period, the total arterial diameter was higher on the operated side (2.40 ± 0.27) than on the contralateral side (2.29 ± 0.23) to a statistically significant extent (p=0.004). In the postoperative period, a decrease in arterial diameter was detected on the operated side (2.08 ± 0.30) compared to the contralateral side (2.22 ± 0.20), also with statistical significance (p<0.001; Table 2). 

**Table 2:** Change in artery diameters before and after surgery (N=100) * p<0.05, a - paired samples t-test, A1-2 - A1-2 segments of anterior cerebral artery, b - Mann-Whitney U test, c - independent samples t-test, ICA - internal carotid artery, M1-2 - M1-2 segments of middle cerebral artery, SD - standard deviation

		Preoperative	Postoperative		Difference %↓
Artery diameter (mm)	Side	Mean±SD	Mean±SD	p-value	Mean±SD
ICA	Operated	3.34±0.49	2.92±0.53	<0.001^a^*	12.5±8.9
	Non-operated	3.14±0.42	3.08±0.40	<0.001^a^*	1.7±4.2
	p-value	0.002^c^*	0.018^c^*		<0.001^b^*
M1-2	Operated	2.07±0.28	1.80±0.31	<0.001^a^*	12.7±9.8
	Non-operated	2.03±0.24	2.01±0.22	0.009^a^*	1.0±4.2
	p-value	0.332^c^	<0.001^c^*		<0.001^b^*
A1-2	Operated	1.78±0.22	1.51±0.23	<0.001^a^*	14.7±12.0
	Non-operated	1.68±0.21	1.54±0.20	<0.001^a^*	7.7±10.0
	p-value	<0.001^c^*	0.397^c^		<0.001^b^*
Total	Operated	2.40±0.27	2.08±0.30	<0.001^a^*	13.2±8.3
	Non-operated	2.29±0.23	2.22±0.20	<0.001^a^*	3.0±4.3
	p-value	0.004^c^*	<0.001^c^*		<0.001^b^*

While not statistically significant (p=0.364), the rate of vasospasm was higher in patients with perioperative temporary clips than in those without (47% vs. 36%). The rate of vasospasm was also higher in patients with perioperative rupture than in those without (56% vs. 33%), which was at the limit of statistical significance (p=0.050). The rate of vasospasm was 32% in discharged patients, 80% in patients who needed care, and 46% in patients who died (p=0.002).

The rate of vasospasm was found to be higher among those with a Fisher grade of III or above than among those with a grade of II or below (51.7% vs. 25%, p=0.014). The rate was also higher in patients with a WFNS score and HH classification of II or above compared to those with a score below II (WFNS: ≥II, 59.6% vs. <II, 20.8%, p<0.001; H&H: ≥II, 50% vs. <II, 21.9%, p=0.014). The Fisher grade, WFNS, and HH classification scores of patients with vasospasm were higher than those of patients without vasospasm. This difference was statistically significant (p<0.05). Among patients presenting with vasospasm, there was no statistically significant difference in terms of age or amount of perioperative bleeding (p>0.05; see Table 3).

**Table 3:** Averages of some continuous variables according to the presence of vasospasm (N=100) * p<0.05 with Mann-Whitney U test, SD - standard deviation, WFNS - World Federation of Neurosurgical Societies

	Vasospasm		p-value
	Yes (n=41), mean±SD	No (n=59), mean±SD	
Age	50.71±13.69	50.15±12.68	0.763
Bleeding (cc)	332.20±214.65	288.14±174.02	0.325
Fisher grade	3.00±1.00	2.31±1.24	0.005^c^*
WFNS grade	2.32±1.21	1.81±1.33	0.002^c^*
Hunt-Hess grade	2.34±1.11	1.97±1.20	0.020^c^*

Compared to patients without vasospasm, patients with vasospasm showed a greater, statistically significant decrease in arterial diameter on both the operated and contralateral sides, ICA, M1-M2, A1-A2, and total arterial diameters (p<0.05). In patients with perioperative rupture, there was a greater decrease in arterial diameter on both the operated and contralateral sides, ICA, A1-A2, and total arterial diameters compared to patients without perioperative rupture. This difference was statistically significant (p<0.05).

The area under the curve was found to be 0.967 (95% CI: 0.931-1); accordingly, the change in arterial diameter (% decrease rate) was found to be statistically significant (p<0.001) for determining the presence of vasospasm. Based on the ROC analysis, the most likely change in arterial diameter on the operated side to indicate the presence of vasospasm was calculated from the available data, where the decrease in total arterial diameter was 13.7%. For the cutoff value of 13.7%, the sensitivity was 100%, the specificity was 91.5%, the positive predictive value was 89.1%, the negative predictive value was 100%, and the overall accuracy was 95% (Table 4). 

**Table 4:** Optimal positive cutoff limit for the rate of decrease in total artery diameter of the operated side in determining the presence of vasospasm (ROC analysis results) AUC - the area under the ROC curve, CI - confidence interval, NPV - negative predictive value, PPV - positive predictive value

	Radiological vasospasm occurrence
Cutoff value	13.7%
AUC (%95 CI)	0.967 (0.931-1)
p-value	<0.001
Sensitivity	100% (41/41)
Specificity	91.5% (54/59)
PPV	89.1% (41/46)
NPV	100% (54/54)
Accuracy	95% (95/100)

## Discussion

Our study aimed to bridge a critical gap in the field of neurosurgery by investigating the utility of CTA in predicting the development of vasospasm in patients with aSAH after surgery. The primary outcomes of our study reveal a statistically significant decrease in arterial diameters, particularly in the ICA, M1-M2, and A1-A2 arteries on the operated side compared to the contralateral side. These findings suggest that CTA can be a valuable tool for monitoring postoperative changes in vessel diameter, potentially identifying patients at a higher risk of developing vasospasm. This information can enable clinicians to take proactive measures, such as closer neurological monitoring and targeted interventions, to prevent or mitigate the impact of vasospasm. Moreover, the ability to predict vasospasm early in the postoperative period can significantly improve patient outcomes. It can lead to timely interventions that reduce the risk of cerebral infarction and its associated morbidity and mortality. Our study lays the foundation for further research in this area. It may contribute to developing standardized protocols for using CTA in the postoperative monitoring of aSAH patients. The ability to predict vasospasm early and take proactive measures based on CTA measurements can improve patient care and reduce the devastating consequences of vasospasm following aSAH.

The main cause of focal cerebral ischemia after SAH is CVS [19]. While rebleeding is the most frightening complication that can develop after aSAH, it has gradually decreased in prominence due to the widespread practice of early surgery. Meanwhile, vasospasm has become the most risky complication of SAH in terms of mortality and morbidity [3,8,9]. Therefore, early recognition of vasospasm is vital. Aneurysmal SAH patients with a history of vasospasm should take measures to prevent vasospasm, especially during the riskiest periods. To reduce morbidity and mortality, healthcare providers must watch patients closely to intervene quickly to treat vasospasm. 

Although the literature reports a lower risk of developing vasospasm among elderly patients, the relationship between age and vasospasm was not statistically significant in the present study (p=0.763). In addition, while some studies evidence that arterial diameters can vary according to age and gender, our study excludes this risk, as it evaluated not only the diameter but also the change [20]. However, we found that the risk of developing cerebral vasospasm was statistically significant in patients with high Fisher (p=0.005), WFNS (p=0.002), and HH (p=0.02) scores. Existing literature has reported similar findings. In addition, some publications have associated intraprocedural bleeding during embolization with vasospasm, which is consistent with our study [21].

Methods such as DSA, TCD, magnetic resonance imaging, CTA, computed tomography perfusion, and magnetic resonance perfusion are used for the imaging of vasospasm. Previous studies have shown that CTA is effective for diagnosing vasospasm, particularly through the evaluation of vasoconstriction and volumetric vessel analysis [22,23]. While CTA has the advantages of being rapid, affordable, widely available, and non-invasive, it also poses limitations, such as ionizing radiation, contrast injection, clip- or coil-induced artifacts, the requirement of transportation to the CT scan, and a fair level of inter-rater reliability [24]. 

Imaging parameters to predict vasospasm and delayed cerebral ischemia (DCI) can be used alone or in combination with clinical markers to increase specificity and sensitivity. To evaluate the risk of DCI, the modified Fisher scale and the WFNS scale have been combined in the VASOGRADE scale, which uses a straightforward, three-category grading system [25]. Another scale that enables risk assessment for in-hospital mortality is based on HH score, age, intraventricular hemorrhage, and rebleed (HAIR) [26]. The VASOGRADE scale and HAIR score did not outperform clinical evaluation in predicting cerebral infarction and a bad prognosis, although they were superior to radiological measurements alone [27,28]. A recent study has developed a four-variable early score for DCI prediction that includes the WFNS scale, the modified Fisher scale, Subarachnoid Hemorrhage Early Brain Edema Score, and intraventricular hemorrhage [29]. However, these studies have reported that these scores are effective for diagnosing vasospasm at the time of diagnosis. 

Our study stands out due to its pioneering approach, as it introduces a novel standardization method for the early diagnosis of patients prone to developing vasospasm or vasoconstriction following postoperative subarachnoid hemorrhage (SAH). Notably, this study is the first to propose such an approach in the existing literature. In our study, the sensitivity was 100% for the cut-off value of 13.7% in early postoperative CTA. The specificity was 91.5%, the positive predictive value was 89.1%, the negative predictive value was 100%, and the overall accuracy was 95%.

Previous research on the use of CTA for the diagnosis of vasospasm has reported that imaging performed after vasospasm develops can effectively support diagnosis [22,23]. Computed tomographic angiography has a sensitivity of about 98% in detecting cerebral aneurysms, and the combination of CT and CTA has a sensitivity of more than 99% in diagnosing aSAH [30]. Other studies of volumetric measurement have aimed to predict the development of cerebral ischemia and its use in the application of treatment modalities. The distinguishing finding of our study is that CTA performed in an early period (<24 hours) before the development of clinical vasospasm can provide insight into the risk of vasospasm development after SAH in patients and may help with early diagnosis, thus providing standardization of diagnosis and treatment. Considering that CTA is a routine test performed in the preoperative and postoperative control periods, our study claims that vasospasm can be predicted without additional imaging modalities. This study is the first to make this suggestion in literature to our knowledge. 

This research has some limitations. The most significant is its retrospective character, as all study data were retrieved from accessible patient files, and no patient interviews or questionnaires were administered. The retrospective screening of CTA is another limiting factor; however, we attempted to mitigate this limitation by having two specialist physicians perform the measurements.

## Conclusions

Vasospasm is one of the most important causes of mortality and morbidity after aSAH, and there is no definitive method to predict the development of vasospasm. By measuring the arterial diameters via CTA, which is an easily accessible method, and comparing them with the cut-off values we have revealed in the study, vasospasm can be predicted, and precautions can be taken accordingly.
